# Epigenetic versus Genetic Deregulation of the KEAP1/NRF2 Axis in Solid Tumors: Focus on Methylation and Noncoding RNAs

**DOI:** 10.1155/2018/2492063

**Published:** 2018-03-13

**Authors:** F. P. Fabrizio, A. Sparaneo, D. Trombetta, L. A. Muscarella

**Affiliations:** Laboratory of Oncology, IRCCS Casa Sollievo della Sofferenza Hospital, San Giovanni Rotondo, Italy

## Abstract

Oxidative and electrophilic changes in cells are mainly coordinated by the KEAP1/NRF2 (Kelch-like erythroid-derived cap-n-collar homology- (ECH-) associated protein-1/nuclear factor (erythroid-derived 2)-like 2) axis. The physical interaction between these two proteins promotes the expression of several antioxidant defense genes in response to exogenous and endogenous insults. Recent studies demonstrated that KEAP1/NRF2 axis dysfunction is also strongly related to tumor progression and chemo- and radiotherapy resistance of cancer cells. In solid tumors, the KEAP1/NRF2 system is constitutively activated by the loss of *KEAP1* or gain of *NFE2L2* functions that leads to its nuclear accumulation and enhances the transcription of many cytoprotective genes. In addition to point mutations, epigenetic abnormalities, as aberrant promoter methylation, and microRNA (miRNA) and long noncoding RNA (lncRNA) deregulation were reported as emerging mechanisms of KEAP1/NRF2 axis modulation. This review will summarize the current knowledge about the epigenetic mechanisms that deregulate the KEAP1/NRF2 cascade in solid tumors and their potential usefulness as prognostic and predictive molecular markers.

## 1. Introduction

Oncogenes and tumor suppressor genes are deregulated in cancer and modify their expression through heterogeneous genetic and epigenetic modifications. All these alterations exert their effects on several cellular processes in which transient modifications of redox balance might occur, such as cell cycle and apoptosis. These transient cellular changes are mainly coordinated by the KEAP1/NRF2 (Kelch-like erythroid-derived cap-n-collar homology- (ECH-) associated protein-1/nuclear factor (erythroid-derived 2)-like 2) signaling pathway [1]. NRF2 is a transcription factor that acts as a master modulator of cellular defense against toxic and oxidative damage, mitochondrial physiology, differentiation, and stem cell maintenance [2–4]. In normal cell conditions, the NRF2 negative regulator KEAP1 forms an ubiquitin ligase complex with cullin 3 (CUL3) and ring-box 1 (RBX1) and targets NRF2 for proteolysis. Upon stress exposure, a specific pattern of KEAP1 cysteine modification arises. By consequence, the KEAP1 releases NRF2 which translocates into the nucleus, where it forms a heterodimeric complex with the small MAF proteins. This complex recognizes the enhancer sequences known as antioxidant response elements (AREs) located in the cytoprotective genes and activates their transcription [5]. Additionally, NRF2 can be subjected to a KEAP1-independent transcriptional and posttranslational regulation, with a consequent alteration of its cellular localization, protein degradation, and DNA-binding capability [6].

Deregulation of the KEAP1/NRF2 axis is actually considered a hallmark in cancer cells, since KEAP1 and NRF2 can modulate oncogenesis, cell proliferation, apoptosis, and tumor cell growth [7].

A decreased levels of KEAP1 protein were firstly reported to be linked to poor outcome in NSCLC patients treated with platinum-based neoadjuvant regimen and in the NSCLC group of patients with lymph node metastases [8, 9].

Actually, the main molecular events that lead to NRF2 abnormal nuclear accumulation in solid tumors can be generally divided into genetic and epigenetic alterations. The final effect is in any case the disruption of protein-protein interaction of the KEAP1/NRF2 crosstalk and its imbalance in expression levels, with a consequent upregulation of cellular detoxifying proteins.

**Figure 1:**
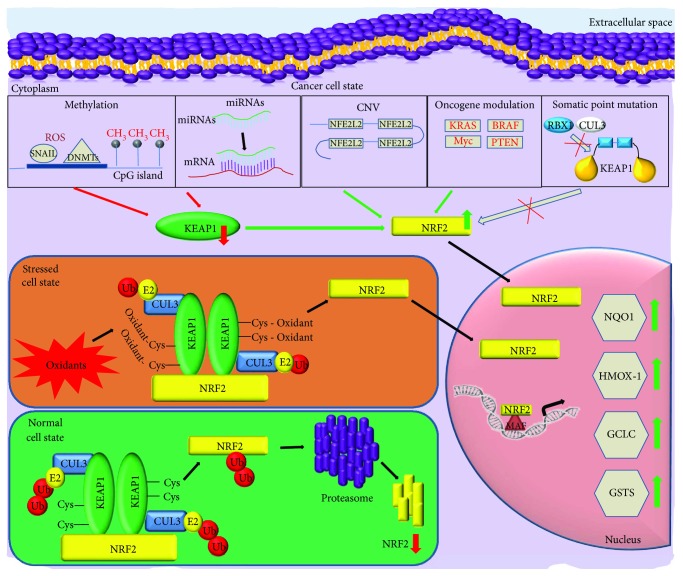
Overview of the main genetic and epigenetic modifications that lead to *KEAP1-NFE2L2* impairment and constitutive NRF2 nuclear accumulation in cancer cells. *NFE2L2* gene copy number variations (CNV), oncogene activity (*KRAS*, *BRAF*, *MYC*, and *PTEN*), DNA promoter methylation, and miRNAs contribute in a synergic manner to increase cancerous NRF2 activity as a result of reduction of *KEAP1* mRNA or increase of *NRF2* mRNA levels and/or protein expression. By contrast, somatic point gain-of-function mutations in *NFE2L2* or in loss-of-function in *KEAP1* promote the disruption of the interaction between KEAP1 and NRF2 and lead the increase of NRF2 protein quantity which translocates into the nucleus.

Genetic alterations were the first reported mechanisms of KEAP1/NRF2 axis deregulation. Biallelic changes of the tumor suppressor *KEAP1* gene (by point mutations and loss of heterozygosity) were described for the first time in NSCLC [10] and, immediately after, in other several malignancies together with *NFE2L2* mutations, the gene that codifies for the NRF2 protein [11]. More recently, the discovery of hypermethylation of the *KEAP1* promoter and noncoding RNAs linked to cell-detoxifying network added a new important dimension in the complex regulation of the KEAP1/NRF2 system (Figure 1) [12].

This minireview describes the recent updates about the deregulation mechanisms of the KEAP1/NRF2 pathway, with a particular focus on the epigenetic modulation of *KEAP1* and *NFE2L2* and their cellular significance and potential impact on cancer patient management.

## 2. The Genetic Deregulation of Keap1/Nrf2 Signaling and Its Translational Impact in Solid Tumors

**Figure 2:**
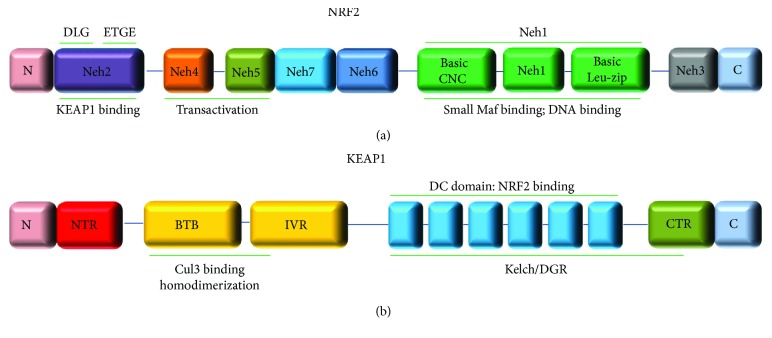
Domain architecture of the NRF2 (a) and KEAP1 (b) proteins. (a) NRF2 protein is divided into seven highly conserved domains, Neh1 to Neh7 (NRF2-ECH homology: Neh). The coordinates of NRF2 protein domains are shown as follows: Neh2 (16-89aa); Neh2 DLG motif (17-32aa), Neh2 ETGE motif (77-82aa), Neh4 (111-134aa), Neh5 (182-209aa), Neh7 (209-316aa), Neh6 (337-394aa), Neh1 (435-568aa), and Neh3 (569-605aa). (b) KEAP1 protein contains a number of functional domains including the N-terminal region (NTR; 1-60aa), broad complex, tramtrack and bric-a-brac (BTB; 61-179aa), the intervening linker domain (IVR; 180-314aa), the double glycine/Kelch domain harboring six Kelch-repeat domains (315-359aa; 361-410aa; 412-457aa; 459-504aa; 506-551aa; 553-598aa), and the C-terminal region (CTR; 599-624aa).

Among the genetic lesions that affect the KEAP1/NRF2 activity, point mutations are the most frequently investigated ones in solid tumors. They commonly occur in the exonic regions that codify for the KEAP1 and NRF2 interaction sites (the Kelch/DGR domain of KEAP1 and the Neh2 domain of NRF2) and induce a general failure of the ubiquitination process led by the KEAP1 (Figure 2).

By consequence, the NRF2 escapes from proteasomal degradation and increases the ARE-target gene expression with an enhancement of the antioxidant defense system and chemo- and radioresistance of cancer cells [13, 14]. Moreover, since the *KEAP1* is able to negatively modulate the BCL-2 and p62 degradation, the *KEAP1* point mutations also lead to an accumulation of these proteins with a general deregulation of apoptosis, autophagy, and inflammation [15, 16].

Loss-of-function mutations of the human *KEAP1* gene have been firstly reported in NSCLC with a prevalence of about 20–25%. These mutations were frequently observed in the lung papillary subtype and in TTF-1 negative lung adenocarcinoma [10, 17, 18]. Moreover, *KEAP1* point mutations were identified in several human cancers such as gastric (11.1%), liver (2–8%), colorectal (7.8%), prostate (1.3%), gallbladder (30.7%), ovarian (37%), glioma (1.7%), head and neck (42%), and clear renal cell carcinoma (4.7%) [19–29]. More recently, Fernandez-Cuesta et al. and Derks reported *KEAP1* genetic alterations as a new uncovered molecular hallmark of LCNEC (lung cancer neuroendocrine) with adenocarcinoma-like features [30, 31]. This last finding was also confirmed by a different group which reported a prevalence of *KEAP1-NFE2L2* (31%) alterations in tumors with high neuroendocrine gene expression, mainly cooccurrent with gene mutations [4, 32, 33].

Gain-of-function *NFE2L2* mutations are generally mutually exclusive with respect to KEAP1 mutations and are frequently located into the DLG or ETGE motifs. *NFE2L2* point mutations were identified in several tumors with squamous histological features, such as esophageal, skin, lung, and laryngeal carcinomas. An increased *NFE2L2* gene copy number was also described in lung squamous cell carcinoma. An increased *NFE2L2* gene copy number was also described in lung squamous cell carcinoma. All of these mutations are generally missense changes that interfere with the KEAP1 ability to bind to NRF2, thus inducing an escape of NRF2 degradation without changing its gene functionality [34–37]. Somatic lesions linked to KEAP1/NRF2 axis deregulation were also reported in the *CUL3* gene, the component of the E3 ligase complex KEAP1/CUL3/RBX1 that marks NRF2 for proteasomal degradation [7, 38]. Mutations in *CUL3*, together with those in *NFE2L2*, are frequent in hereditary type 2 papillary renal cell carcinoma (PRCC2). In squamous carcinoma of the head and neck, somatic lesions of *CUL3*/*NFE2L2/KEAP1* have a prevalence of 64% and were associated with a lower patient overall survival (Tables 1 and 2) [25, 39–41].

Dysfunction of the KEAP1/NRF2 axis by genetic mutations is gradually becoming a milestone to understand cancer development, progression, and resistance to conventional and biological treatments [42]. It is now well known that loss-of-function mutations of the *KEAP1* gene or gain-of-function mutations in *NFE2L2* enhance the resistance of cancer cells to anticancer drugs, such as etoposide and carboplatin, and it is associated with poor outcome of platinum-based advanced NSCLC patients [43]. Nuclear accumulation of NRF2 was also correlated with a poor survival of lung SqCC and pancreatic adenocarcinomas and a worse progression free survival (PFS) in patients treated with surgery only [44–47]. Aberrant NRF2 activation due to *KEAP1* alterations is also reported as one of the molecular mechanisms of chemoresistance of gallbladder cancer under 5-FU-based regimen and of colorectal cancer under demethylase and methyltransferase treatments [20, 48, 49].

Jeong and coworkers suggested a new role for *KEAP1* and *NFE2L2* mutations in radiotherapy resistance of NSCLC patients and in identifying patients who might benefit from radiation dose escalation [50]. Knockdown experiments reported that radiochemosensitization was led by CDK20 that competes with NRF2 for KEAP1 binding and induces nuclear translocation of NRF2 and the enhancement of its transcriptional activity. This ultimately results in proliferation defects and provides new insights into the cellular response to NRF2-mediated DNA damage [51].

An interesting link between the KEAP1/NRF2 axis and target therapies was recently reported. Cell proliferation in cancer was demonstrated to be cross-regulated by KEAP1/NRF2 and EGFR signaling. Moreover, cells expressing oncogenic allele of *KRAS* are able to activate NRF2 via the MAPK pathway in mouse embryonic fibroblasts [52, 53]. In the same way, the loss of *KEAP1* by the CRISPR-Cas9 system cooperates with the tumor mutational landscape in modulating the response to BRAF, MEK, EGFR, and ALK inhibition and in allowing cancer cells to increase their ability to resist to treatments and proliferate [54]. KRAS activity confers in NSCLC chemoresistance also by upregulating NRF2 through the link with TPA response element (TRE) located in exon 1 of the *NFE2L2*. In the same context of resistance to target therapy, it is possible that *NFE2L2* mutations can contribute to survival under crizotinib treatment and can allow the cells to acquire additional resistance mutations over time. In line with these hypotheses, Krall et al. recently identified a hotspot mutation in *NFE2L2* in a patient with acquired resistance to ALK inhibitors that could exert a synergic effect with a secondary *ALK* mutation in the resistance to second-generation ALK inhibitors [55].

Recent additional studies in this field gave the first hint of the prognostic role of single-nucleotide polymorphisms (SNPs) of the *KEAP1* gene in breast cancer without inducing any evident and detectable variations of the protein structure or conformation. More specifically, five tagging SNPs (rs34197572, rs9676881, rs1048290, rs11085735, and rs8113472) located in the *KEAP1* were genotyped and appeared to be in allelic linkage disequilibrium (LD) with each other. This finding suggests the existence of a haplotype block at the *KEAP1* gene locus that might correlate with specific clinical features of cancer patients [56]. The two SNPs rs9676881 and rs1048290 resulted to be significantly associated with a shorter PFS survival in invasive breast cancer patients. The main hypothesis is that they reside into cell type-specific regulatory elements that modulate the binding capability of critical transcriptional factors, which in turn change target gene expression. Thus, it might explain the observed correlation with the high KEAP1 protein expression levels and the high cytoplasmic localization of NRF2 in breast tissues [57]. The SNP rs1048290 is located in the DGR domain, so it may affect the maintenance of physiological levels of NRF2.

Tumor susceptibility SNPs might be also associated with specific miRNA/lncRNA binding regions [58, 59]. The SNP rs1048290 was found in LD with the SNP rs9676881, which is located in a putative enhancer region, few bases downstream of the 3′-untraslated region (3′-UTR) of the *KEAP1* gene and 410 bp from the miR-200a binding site. By consequence, it is clear to suppose that a LD may exist with these silent variations and the specific miRNA binding site. However, the role of *KEAP1* SNPs in predicting patient survival remains controversial. The two SNPs rs9676881 and rs1048290 appeared to be the most interesting ones and resulted to be significantly associated with a shorter PFS survival in both invasive and ER-positive tamoxifen-treated invasive breast cancer patients [60].

## 3. The Aberrant Methylation of the Keap1-Nrf2 Axis and Its Translational Impact in Solid Tumors

Gene promoter hypermethylation at the specific CpGs and chromatin remodeling are two of the main epigenetic events that can modulate gene expression by spatial interfering with the ability to work with the transcriptional machinery.

Epigenetic mechanisms are clearly implicated in the complex regulation of the KEAP1/NRF2 axis and are actually considered the most frequent mechanisms of *KEAP1* silencing in solid tumors [61]. The first hypothesis of an epigenetic dysregulation of the *KEAP1* gene comes from the intriguing observation that the *KEAP1* mutations were not frequent enough to justify alone the frequency of aberrant NRF2 nuclear accumulation reported in lung tumor cells [8]. All the scientific findings on the hypermethylation of the *KEAP1* promoter and its effects on the KEAP1/NRF2 pathway are summarized in Table 3. The first report in this field was in human NSCLC and prostate DU-145 cancer cell lines. The promoter CpGs affected by this phenomenon are grouped into one island located at the P1 region of *KEAP1*, near the transcriptional start site [62–64]. The *KEAP1* promoter hypermethylation was described in neoplastic tissues of patients affected by glioma, breast cancer (51%), and primary NSCLC (47%). In lung cancer, the presence of epigenetic abnormalities in the *KEAP1* gene plus its point mutations/LOH matched with the prevalence of NRF2 nuclear accumulation in NSCLC tissues and was associated with an increased risk of lung cancer progression in surgically resected patients [65–67]. In clear renal cell carcinoma (ccRCC), the epigenetic modulation of *KEAP1* was shown to be the leading mechanism of *KEAP1* deregulation (48.6%), thus supporting a driver role of the KEAP1/NRF2 axis in renal cancer. TCGA (The Cancer Genome Atlas) concomitant data analysis suggested that *KEAP1* hypermethylation is able to strongly predict patient survival [68]. In primary breast cancers and preinvasive lesions, an aberrant *KEAP1* promoter methylation was seen to be more recurrent in ER-positive, HER2-negative than in triple-negative breast cancers and was hypothesized to be a prognostic marker since a higher mortality risk in triple-negative patients was predicted. Moreover, *KEAP1* promoter silencing by methylation was also predictive of a lower risk of tumor relapse in patients treated with sequential therapy of anthracyclines and cyclophosphamide followed by taxanes [67]. Gliomas are the second tumor described by our group to have a promoter hypermethylation. In these tumors, it was reported that *KEAP1* epigenetic modifications were associated with poor prognosis and contribute to the prediction of the disease progression of patients subjected to radiotherapy and temozolomide treatment [65]. The role of KEAP1/NRF2 in radiation has been also elucidated in A549 lung adenocarcinoma cell line under DNMT inhibitor genistein treatment. The pharmacological demethylation of the *KEAP1* CpG promoter islands was demonstrated to induce an increase in transcript levels and a consequent overexpression of NRF2, GSS, and HO-1 [69].

Aberrant *KEAP1* methylation was also reported in 53% of colorectal cancer and head and neck cancer tissues (29.3%) and was also linked to the worse prognosis of these tumors [70, 71]. In pancreatic cancer cell lines, the suppression of KEAP1 protein was demonstrated to be correlated with UHRF1, a scaffold protein for DNA methyltransferase DNMT1 [72].

A possible role of epigenetic variations in the modulation of NRF2 expression is less investigated. Li and colleagues showed that a low methyltransferase EZH2 expression correlates both lung cancer cell lines and tissues with an elevated expression of NRF2, NQO1 (NAD(P)H-quinone oxidoreductase 1), and HO-1 (heme oxygenase 1). Since the EZH2 is involved in the establishment and/or maintenance of chromatin architecture and histone methylation, its downstream effect was attributed to a decrease in the trimethylation of lysine 27 on histone H3 (H3K27Me3) in the *NFE2L2* promoter region [73]. Recently, Kang and colleagues focused on the causative relationship between NRF2 expression and its epigenetic alterations, especially in the context of DNA methylation at cytosines and histone methylation status during 5-fluorouracil- (5-FU-) induced oxidative stress in colon cancer cells. They found that elevated reactive oxygen species (ROS) level induced by 5-FU activates TET (ten-eleven translocation) DNA demethylases and produces a hypomethylation of the *NFE2L2* promoter with consequent activation of NRF2 translation. This, in turn, upregulates the expression of the antioxidant enzymes and generates the resistance to 5-FU in cancer cells [48, 49].

## 4. MicroRNAs Directly Targeting the Keap1/Nrf2 Pathway

An intriguing epigenetic way of KEAP1/NRF2 pathway deregulation in tumor cells comes from miRNAs that act in cancer as oncogenes or tumor suppressors [74]. miRNAs are proximately 22 nucleotide single-stranded noncoding RNA molecules which regulate gene expression at posttranscriptional levels by binding to the 3′-untranslated regions (UTRs) of specific mRNAs. They generally affect the translation or stability of mRNA molecules through the interaction of specific mRNAs with complementary base pairing.

**Figure 3:**
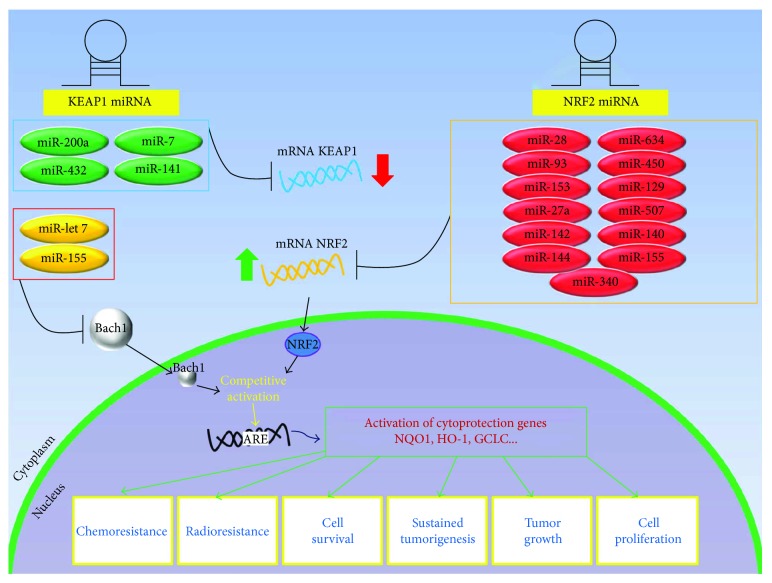
Left and right panels show how miRNA modifications may contribute to down and upregulate the KEAP1/NRF2 signaling in cancer. Representative scheme on the left side summarizes a group of miRNAs that directly target *KEAP1* mRNA and indirectly impact on the transcriptional activity of the NRF2 into the nucleus. Other miRNAs modulate BACH1, a transcription factor that competes with NRF2 leading to the link at the antioxidant response element (ARE) of detoxifying genes. The schematic model on the right side depicts those miRNAs that directly target *NFE2L2* and impact on the general mRNA and protein levels of NRF2 and, by consequence, on the activation of detoxification NRF2 target genes with a great impact on chemo- and radioresistance, survival, growth, and proliferation of tumor cells.

Two different blocks of miRNAs can be distinguished in the context of posttranscriptional regulation of the KEAP1/NRF2 pathway (Table 4) [75]. The first group includes the miRNAs that directly target *NFE2L2* and usually negatively regulate the KEAP1/NRF2 pathway by decreasing the NRF2 protein levels. The second one comprises those miRNAs that directly interact with *KEAP1* and indirectly influence the NRF2 signaling. In addition, a lot of miRNAs are reported to indirectly modulate the ARE-mediated redox signaling through their interaction with additional factors located in the crossing points of the NRF2 network (Figure 3). All of these interactions are complex and still remain to be fully elucidated. So we focalized our attention on the main findings concerning NRF2 and KEAP1.

Several miRNAs predicted to target the KEAP1/NRF2 axis were identified by bioinformatic analysis of miRNA databases [76]. Some of these were experimentally proven to directly target and repress the NRF2 activity. The negative effects on the NRF2 expression by miR-28 were firstly documented in MCF-7 breast cancer cells [77]. A similar activity was described for miR-155 in 16-HBE human bronchiolar epithelial cells under arsenite treatment, together with an observed upregulation of glutathione (GSH), nitric oxide (NO), and superoxide dismutase (SOD) [78]. The increased expression of miR-155 correlates with radiation-induced severe fibrosis in a murine skin model [79], similar to miR-140, whose deficiency increased activation of TGF-*β*1 signaling, inflammation, and myofibroblast differentiation in fibrotic lung tissue after radiation treatment [80, 81]. Among functionally validated miRNAs that regulate NRF2, miR-144 recently emerged as having a central role in the modulation of cellular stress response in blood malignancies and solid tumors. In K562 cell line and primary erythroid progenitor cells, it was seen that increased levels of miR-144 were associated with reduced NRF2 levels in HbSS reticulocytes. By contrast, inhibition of miR-144-3p in human leukemia HL-60 cells reduced cell viability and prompted apoptosis by interfering with NRF2 activity. In hepatocellular carcinoma cell lines, miR-144 overexpression was reversely correlated with an enhancement of 5-fluorouracil- (5-FU-) induced cytotoxicity and apoptosis and with GSH biosynthesis in neuroblastoma SH-SY5Y cells via NRF2 [82–84].

A direct effect of miR-93 on nuclear accumulation of NRF2 was well described by Singh et al. using a rat model of breast carcinogenesis. A significant reduction in carcinogenesis-associated phenotypes such as mammosphere development, antiapoptosis, and DNA damage was observed [85].

In silico analysis and *in vitro* studies on different sets of tumor cell lines recently provided more additional insights into the role of KEAP1/NRF2 axis modulation by miRNAs. In SH-SY5Y neuroblastoma cells, the 3′-UTR of *NFE2L2* is targeted by miR-153, miR27a, and miR-142-5p, with a consequent decrease in Gclc glutamate-cysteine ligase (GCLC) and glutathione-disulfide reductase (GSR) expression levels [86]. The functional impact of miR-153-3p/NRF2 interaction was firstly reported in breast cancer cell lines and recently highlighted by microarray studies in oral squamous cell carcinoma cell lines and tissues. Low expression levels of miR-153-3p significantly correlate with tumor cell migration and invasion [87, 88]. In the same histological pattern of esophageal cell carcinoma, miR-340 was shown to directly modulate the NRF2 expression levels, thus interfering with the chemoresistance phenotype under cisplatin treatment [89]. Finally, miR-507, miR-634, miR-450a, and miR-129-5p appeared to negatively modulate the NRF2 activity by targeting both the *NFE2L2* and *ME1* transcripts, a well-known target of NRF2 [90]. In NSCLC A549 cells, this group of miRNAs exerts a synergic effect in increasing sensitivity of cell growth suppression under cisplatin treatment [91].

By looking at the KEAP1 regulation side, miR-141 was the first reported miRNA to target *KEAP1* by binding to its 3′-UTR sequence site in ovarian carcinoma cell lines [92]. The upregulation of miR-141 expression decreases the 5-FU-mediated effects and apoptosis in hepatocellular carcinoma cell lines by inducing nuclear translocation of NRF2 and activation of *HO-1* gene transcription [93]. A direct inhibition effect of miR-200a on *KEAP1* was elucidated in human MDA-MB-231 and Hs578T breast and esophageal squamous cell carcinoma cells under methylseleninic acid (MSA) treatment. MSA acts as a chemopreventive agent that is able to induce miR-200a expression and inhibits *KEAP1* through the Krüpple-like factor 4 (*KLF4*) [60, 94, 95]. Interesting results came from the investigation by Hartikainen et al. In their work, SNP rs1048290 has been found in LD with SNP rs9676881, which is located in a putative enhancer region, few bases downstream of the 3′-untranslated region (3′-UTR) of the *KEAP1* gene, the specific target region of miR-200a [56]. More recently, a direct action of miR-7 on KEAP1 expression was described in the human neuroblastoma cells. By targeting the 3′-UTR of *KEAP1* mRNA, miR-7 enhances the nuclear localization of NRF2 and induces an increased expression of HO-1 and glutamate-cysteine ligase modifier subunit (GCLM). The control of cell survival under stress by miR-7 was amplified by the observed variation of intracellular hydroperoxide levels and increases in the reduced form of glutathione levels [83, 96]. A similar effect was described for miR-196 in human hepatoma cells against hepatitis C virus infection [97]. The CRISPR/Cas9 system was used to prove the direct binding to the coding region of *KEAP1* by miR-432-3p in the esophageal squamous cell carcinoma (ESCC). In this tumor, miR-432-3p overexpression correlates with a downregulation of the KEAP1 expression, thus inducing a decrease in the sensitivity of tumor cells to cisplatin and other chemotherapy drugs [98].

A lot of miRNAs were also reported to regulate the KEAP1/NRF2 pathway independently from the KEAP1 or NRF2 activity. The *let-7* family modulates the DICER expression and represents the first example of cancer regulation by miRNA in humans [99]. Not less importantly, *let-7* showed to inhibit the expression of several oncogenes involved in cellular proliferation, such as *RAS* (rat sarcoma), *MYC* (avian myelocytomatosis viral oncogene homolog), and *HMGA2* (high-mobility group AT-hook 2) [100, 101]. The miRNAs let-7b and let-7c were firstly demonstrated to negatively modulate the expression of BACH1 in the liver, a transcription factor that works in association with the small MAF proteins in a dominant condition in respect of NRF2 [102, 103]. By consequence, the repression of BACH1 induces an upregulation of HO-1 expression via NRF2 transcription [104].

## 5. Long Noncoding RNAs Linked to the Keap1/Nrf2 Pathway

Long noncoding RNAs (lncRNAs) are non-protein-coding transcripts longer than 200 nucleotides which are expressed in a sense, antisense, or bidirectional manner. Different to protein-coding genes, they show a high density of DNA methylation around their transcription start sites, independent of their expression status [105]. A growing number of evidences elucidated the role of lncRNAs in the initiation, progression, and stem cell pluripotency of cancer cells [106]. Little is known about the role of lncRNAs in the modulation of the detoxification processes of cells.

The most recent findings are those related to smoke and cancer-associated lncRNA 1 (SCAL1) and lncRNA regulator of reprogramming (ROR). The SCAL1 is the first characterized long noncoding RNA activated by NRF2 and is considered one of the downstream mediators of NRF2-induced oxidative stress protection in airway epithelial cells. Under stress induced by cigarette smoke, the SCAL1 expression increases in lung cancer cell lines and appears to be directly correlated with *NFE2L2* mutations [107]. In human bronchial epithelial cells, a knockdown gene approach revealed that NRF2 can regulate the expression level of SCAL1 by binding to the nuclear factor erythroid-derived 2 (NF-E2) motif located in the promoter region of its gene [108]. Conversely to SCAL1, Zhang and colleagues proved that in breast cells, NRF2 controls the ROR lncRNA expression by binding two different NRF2 response elements flanking the *ROR* promoter region. *NFE2L2* knockdown leads to the overexpression of lncRNA ROR in mammary embryonic stem cells [109].

## 6. Concluding Remarks

Significant advances have been made in these last years to understand the regulation mechanisms of the KEAP1/NRF2 system. However, although KEAP1/NRF2 dysfunction is now well known to confer resistance to chemo- and radiotherapy, the *KEAP1-NFE2L2* mutational status assessment is not used to make treatment decisions in lung cancer yet. Moreover, molecular profiling of these two proteins in pretreated and resistant tumor samples will help to elucidate if the loss of *KEAP1* or the gain of *NFE2L2* may be clinically relevant mechanisms of acquired and intrinsic resistance to therapies in lung cancer and other solid tumors or not. A general limitation to clarify these issues remains and consists of a lack of available rebiopsy tissue specimens.

From an epigenetic point of view, the effects produced by *KEAP1* hypermethylation on the KEAP1/NRF2 signaling in cancer remain partially understood. Firstly, it is natural to wonder what the real role of P1 CpG island methylation is and if the methylation status of *KEAP1* exclusively affects its expression or could additionally interfere with the ability to bind to NRF2 in promoting tumor progression and resistance to therapies. According to these observations, it would be of great interest to determine if in tumors with different origins there are similar or different methylation CpG density patterns at the P1 region and if demethylation of the *KEAP1* promoter in neoplastic tissues could really suppress tumor progression and enhance resistance to therapies.

Given that posttranscriptional modifications play important roles in regulating the stability and translation of mRNAs, more studies on the regulation of the KEAP1/NRF2 pathway by miRNAs will corroborate their key roles in clinical practice. However, this approach will require a greater knowledge of how drug treatment influences miRNA expression and how miRNA expression could influence the multifaced KEAP1/NRF2 network.

## Figures and Tables

**Table 1:** Functionally investigated *KEAP1* gene mutations in tumor tissues and cell lines.

Cancer tissue or cell line types	Nucleotide change	Amino acid change	Mutation type/clinical prediction	Protein domain	Functional effects of *KEAP1* mutant
Breast cell line and ductal carcinoma	c.68G>A	p.C23Y	Missense/pathogenic	NTR	Repression of NRF2-dependent transcription activity and ubiquitination defects
Lung AC, lung SCC	c.212G>T	p.R71L	Missense/pathogenic	BTB	Wild-type behavior
Stomach AC	c.246G>T	p.Q82H	Missense/pathogenic	BTB	Impaired effect on NRF2 pathway activation
EOC	c.319T>C	p.F107L	Missense/pathogenic	BTB	Enhance the NRF2 nuclear localization and its transcription activity
BUC, EOC	c.347G>C	p.R116P	Missense/pathogenic	BTB	Enhance the NRF2 nuclear localization and its transcription activity
Lung AC, lung SCC	c.463G>T	p.V155F	Missense/pathogenic	BTB	Enhance the binding of KEAP1 to NRF2 and facilitate NRF2 ubiquitination
EOC	c.475G>A	p.A159T	Missense/pathogenic	BTB	Enhance the NRF2 nuclear localization and its transcription activity
Lung AC, lung SCC	c.499G>T	p.V167F	Missense/pathogenic	BTB	Weakly affect the bind of KEAP1 to NRF2 without suppressing the NRF2 activity
BTC	c.543_544insC	p.S181fs^∗^11	Frameshift/NS	IVR	Induce the loss of KEAP1 repression activity on NRF2
HCC	c.548A>G	p.N183S	Missense/pathogenic	IVR	Induce an impaired binding of KEAP1 to the CUL3 ubiquitin ligase
Lung AC	c.556G>C	p.G186R	Missense/pathogenic	IVR	Induce an enhanced binding of KEAP1 to NRF2 and facilitate its ubiquitination without suppressing NRF2-mediated transcription
EOC	c.563C>T	p.A188V	Missense/pathogenic	IVR	Enhance activation of NRF2 pathway and an increase of its transcriptional activity and nuclear localization
Lung AC	c.599A>C	p.H200P	Missense/pathogenic	IVR	Wild-type behavior
Lung SCC	c.671C>A	p.S224Y	Missense/pathogenic	IVR	Wild-type behavior
Lung SCC	c.691C>G	p.L231V	Missense/pathogenic	IVR	Wild-type behavior
Stomach AC	c.698G>A	p.S233N	Missense/pathogenic	IVR	Induce an impaired binding of KEAP1 to the CUL3 ubiquitin ligase
CESC, colorectal AC	c.700C>T	p.R234W	Missense/pathogenic	IVR	Induce an impaired binding of KEAP1 to the CUL3 ubiquitin ligase
Lung AC	c.706G>C	p.D236H	Missense/pathogenic	IVR	Reduce KEAP1-mediated repression of NRF2
Lung SCC, liver	c.706G>T	p.D236Y	Missense/pathogenic	IVR	Induce an impaired binding of KEAP1 to the CUL3 ubiquitin ligase
Lung AC	c.711delG	p.L237fs^∗^1	Frameshift (stop codon)/NS	IVR	Reduce KEAP1-mediated repression of NRF2
Lung AC	c.724G>A	p.E242K	Missense/pathogenic	IVR	Induce an impaired binding of KEAP1 to the CUL3 ubiquitin ligase
Lung SCC	c.728C>G	p.S243C	Missense/pathogenic	IVR	Induce an enhanced binding of KEAP1 to NRF2 and facilitate its ubiquitination without suppressing NRF2-mediated transcription
BUC, lung AC	c.730G>A	p.E244K	Missense/pathogenic	IVR	Induce an impaired binding of KEAP1 to the CUL3 ubiquitin ligase
BTC, stomach	c.746G>A	p.C249Y	Missense/pathogenic	IVR	Mutant *KEAP1* fails to repress NRF2-dependent transactivation
Breast AC	c.767A>G	p.D256G	Missense/pathogenic	IVR	Induce an impaired binding of KEAP1 to the CUL3 ubiquitin ligase
PF	c.790G>A	p.V264I	Missense/pathogenic	IVR	Reduce KEAP1-mediated repression of NRF2
Lung AC	c.814C>T	p.R272C	Missense/pathogenic	IVR	Reduce KEAP1-mediated repression of NRF2 (impaired Nrf2 degradation)
Stomach AC	c.838T>C	p.F280L	Missense/pathogenic	IVR	Induce an impaired binding of KEAP1 to the CUL3 ubiquitin ligase
Stomach AC	c.842T>C	p.L281P	Missense/pathogenic	IVR	Induce an impaired binding of KEAP1 to the CUL3 ubiquitin ligase
Lung AC, lung LCC	c.851A>T	p.Q284L	Missense/pathogenic	IVR	Reduce KEAP1-mediated repression of NRF2
Stomach AC	c.863G>A	p.C288Y	Missense/pathogenic	IVR	Induce and impair binding of KEAP1 to NRF2 and facilitate NRF2 ubiquitination and its degradation
Lung AC, liver	c.880G>T	p.D294Y	Missense/pathogenic	IVR	Lead to deleterious effect on protein stability
Lung SCC	c.953C>T	p.P318L	Missense/pathogenic	KELCH1	Wild-type behavior
Lung SCC	c.?	p.P318_fs	Frameshift/NS	KELCH1	Impact on the KEAP1-NRF2 association and NRF2 degradation
EOC	c.1234C>T	p.P319S	Missense/NS	KELCH1	Enhance activation of NRF2 pathway and an increase of its transcriptional activity and nuclear localization
Lung SCC	c.959G>A	p.R320Q	Missense/pathogenic	KELCH1	Induce an enhanced binding of KEAP1 to NRF2 and facilitate its ubiquitination without suppressing NRF2-mediated transcription
Lung AC, lung SCC	c.965C>T	p.P322L	Missense/pathogenic	KELCH1	Lead to deleterious effects on KEAP1 protein stability
Colorectal AC	c.989C>T	p.T330I	Missense/pathogenic	KELCH1	Impair the KEAP1 binding to NRF2 through the KEAP1 DC pocket (lower affinity)
Lung AC	c.994G>T	p.G332C	Missense/pathogenic	KELCH1	Induce an enhancement of the NRF2 activity
Gallbladder	c.996_996delC	p.G332fs^∗^67	Frameshift/NS	KELCH1	Lead a loss of NRF2 repression by KEAP1
Lung AC	c.997G>T	p.G333C	Missense/pathogenic	KELCH1	Induce misfolding effects and decrease the KEAP1 stability and capability to bind NRF2
Lung	c.1001>T	p.Y334F	Missense/NS	KELCH1	Disrupt the integrity of the Kelch domain of KEAP1
HCC	c.1007G>A	p.R336Q	Missense/pathogenic	KELCH1	Impair the KEAP1 binding to NRF2 through the KEAP1 DC pocket (lower affinity)
Gallbladder AC, breast AC	c.1013C>T	p.S338L	Missense/pathogenic	KELCH1	Mutant *KEAP1* fails to repress NRF2-dependent transactivation
HCC	c.1024C>A	p.L342M	Missense/pathogenic	KELCH1	Impair the KEAP1 binding to NRF2 through the KEAP1 DC pocket (lower affinity)
Lung AC	c.1036InsT	p.S346_fs	Frameshift (stop codon)/pathogenic	KELCH1	Lead to a premature termination and truncated KEAP1 protein
PF	c.1043insG	p.348_fs	Frameshift (stop codon)/NS	KELCH1	Result in a frameshift and produce a truncated KEAP1 protein, with a lower KEAP1-mediated repression of NRF2
Lung AC, stomach cell lines	c.1048G>A	p.G350S	Missense/NS	KELCH1	Reduce KEAP1-mediated repression of NRF2
Prostate	c.1069G>A	p.D357N	Missense/pathogenic	KELCH1	Impair the KEAP1 binding to NRF2 through the KEAP1 DC pocket (lower affinity)
Colorectal AC	c.1075C>T	p.Q359X	Nonsense/pathogenic	KELCH1	Impair the KEAP1 binding to NRF2 through the KEAP1 DC pocket (lower affinity)
Lung AC samples and cell lines	c.1090G>T	p.G364C	Missense/pathogenic	KELCH2	Abolish the KEAP1-NRF2 interaction
Lung AC	c.GCC1098TTA	p.L367_fs	Frameshift/pathogenic	KELCH2	Produce a truncated KEAP1 protein
Lung AC	c.1106T>C	p.V369A	Missense/pathogenic	KELCH2	Lead to deleterious effects on KEAP1 protein stability
Gallbladder AC, HCC	c.1136G>A	p.G379D	Missense/pathogenic	KELCH2	Mutant *KEAP1* fails to repress NRF2-dependent transactivation. Induce a misfolding effects on the KEAP1 protein and decrease its ability to bind NRF2
Lung AC	c.1238G>T	p.L413R	Missense/pathogenic	KELCH3	Mutant *KEAP1* fails to repress NRF2-dependent transactivation. Induce misfolding effects on the KEAP1 protein and decrease its ability to bind NRF2
Lung AC	c.DelGG?	p.L413_fs	Frameshift (stop codon)/NS	KELCH3	Reduce KEAP1-mediated repression of NRF2
Lung AC	c.1243C>G	p.R415G	Missense/pathogenic	KELCH3	Affect the ability of KEAP1 to repress NRF2 and lost the ability to bind and sequester NRF2 in the cytoplasm
Lung SCC, ESCC	c.1264G>A	p.D422N	Missense/pathogenic	KELCH3	Enhance the binding of KEAP1 to NRF2 and facilitate NRF2 ubiquitination
Lung AC, lung SCC	c.1268G>T	p.G423V	Missense/pathogenic	KELCH3	Enhance the binding of KEAP1 to NRF2 and facilitate NRF2 ubiquitination
Lung AC	c.1280C>T	p.A427V	Missense/pathogenic	KELCH3	Reduce KEAP1*-*mediated NRF2 repression ability but Kelch domain should still be able to interact effectively with the ETGE and the DLG sites of NRF2
Lung AC	c.1288G>T	p.G430C	Missense/pathogenic	KELCH3	Induce a misfolding effect on the KEAP1 protein and decrease its ability to bind NRF2 and sequester NRF2 in the cytoplasm.
Lung AC cell line	c.1329T>G	p.Y443_fs	Frameshift (stop codon)/NS	KELCH3	Reduce KEAP1-mediated repression of NRF2
Lung AC	c.1370delG	p.L457fs^∗^1	Frameshift (stop codon)/NS	KELCH3	Result in a frameshift and produce a truncated KEAP1 protein, with a lower KEAP1-mediated repression of NRF2
Lung AC	c.1396G>C	p.A466P	Missense/pathogenic	KELCH4	Lead to deleterious effects on KEAP1 protein stability
Lung SCC	c.?	p.N469fs	Frameshift/NS	KELCH4	Impact on the KEAP1-NRF2 association and the KEAP1 ability to suppress NRF2
Lung AC, lung SCC, ESCC, UADT	c.1408C>T	p.R470C	Missense/pathogenic	KELCH4	Exhibit enhanced binding to NRF2 and facilitate NRF2 ubiquitination
Lung LCC	c.1426G>A	p.G476R	Missense/NS	KELCH4	Induce a misfolding effect on the KEAP1 protein and decrease its ability to bind NRF2 and sequester NRF2 in the cytoplasm.
Lung SCC	c.1438G>T	p.G480W	Missense/pathogenic	KELCH4	Reduce the KEAP1-NRF2 binding
Lung AC	c.1448G>A	p.R483H	Missense/pathogenic	KELCH4	Reduce the KEAP1-NRF2 binding
Lung AC	c.1477G>C	p.E493Q	Missense/pathogenic	KELCH4	Induce an upregulation of the NRF2 activity
Lung SCC	c.1632G>T	p.W544C	Missense/pathogenic	KELCH5	Reduce the KEAP1-NRF2 binding
Liver, lung AC	c.1661G>A	p.R554Q	Missense/pathogenic	KELCH6	Reduce the KEAP1-NRF2 binding
Liver	c.1662G>A	p.W554X	Missense/pathogenic	KELCH6	Decrease the NRF2 repression activity
PF	c.1663_1680del18	p.S555_T560del	In frame/NS	KELCH6	Reduce KEAP1-mediated repression of NRF2
Lung AC	c.1709G>T	p.G570V	Missense/pathogenic	KELCH6	Reduce the KEAP1-NRF2 binding
Lung AC	c.1772G>T	p.W591L	Missense/pathogenic	KELCH6	Impair the KEAP1 binding to NRF2 through the KEAP1 DC pocket (lower affinity)
Colorectal AC	c.1816G>A	p.V606M	Missense/pathogenic	CTR	Induce an upregulation of the NRF2 activity
EOC	c.1831G>A	p.E611K	Missense/pathogenic	CTR	Enhance activation of NRF2 pathway and an increase of its transcriptional activity and nuclear localization

Cosmic (Catalogue of Somatic Mutations in Cancer) database IP **(**http://cancer.sanger.ac.uk/cosmic/gene/analysis?ln=KEAP1#variants). AC: adenocarcinoma; BUC: bladder urothelial carcinoma; CESC: cervical squamous cell carcinoma; DC: C-terminal *β*-propeller domain; EOC: epithelial ovarian cancer; ESCC: esophageal squamous cell carcinoma; HCC: hepatocellular carcinoma; LCC: large cell carcinoma; PF: pleural fluid; SCC: squamous cell carcinoma; UADT: upper aerodigestive tract; NS: not specified.

**Table 2:** Functional investigated *NFE2L2* gene mutations in tumor tissues and cell lines.

Cancer tissue or cell line types	Nucleotide change	Amino acid change	Mutation type/clinical prediction	Protein domain	Functional effects of *NFE2L2* mutant
HCC	c.68T>G	p.L23R	Missense/pathogenic	Neh2 DLG	Disrupt the intramolecular interactions for KEAP1-DLGex binding and are associated with the strongest increase of NRF2 activity
PRCC, ESCC	c.70T>C	p.W24R	Missense/pathogenic	Neh2 DLG	*NFE2L2* mutant with gain-of-function activity, which is partly resistant to KEAP1-mediated inhibition
Lung SCC, SCLC, ESCC	c.72G>C	p.W24C	Missense/pathogenic	Neh2 DLG	Affect the binding to the KEAP1 dimer and inhibit the KEAP1-mediated degradation of NRF2 and a stronger increase of the NRF2 activity
ESCC	c.76C>G	p.Q26E	Missense/pathogenic	Neh2 DLG	*NFE2L2* mutant with gain-of-function activity, which is partly resistant to KEAP1-mediated inhibition
Lung SCC	c.77A>T	p.Q26L	Missense/pathogenic	Neh2 DLG	Only present in TCGA report
Lung SCC	c.77A>C	p.Q26P	Missense/pathogenic	Neh2 DLG	Only present in TCGA report
HCC	c.78A>C	p.Q26H	Missense/pathogenic	Neh2 DLG	Lead to an activation of NRF2 signaling
Lung SCC	c.79G>C	p.D27H	Missense/pathogenic	Neh2 DLG	Only present in TCGA report
ESCC	c.79G>T	p.D27Y	Missense/pathogenic	Neh2 DLG	Block proper KEAP1-NRF2 binding and inhibit the KEAP1-mediated degradation of NRF2
Lung SCC, ESCC	c.83T>C	p.I28T	Missense/pathogenic	Neh2 DLG	Lead to weaker KEAP1-binding DLG region and inhibit the KEAP1-mediated degradation of NRF2
HCC, lung AC, SCC-UADT	c.85G>C	p.D29H	Missense/pathogenic	Neh2 DLG	Lead and increase NRF2 levels
Lung SCC	c.85G>A	p.D29N	Missense/pathogenic	Neh2 DLG	Inhibit the KEAP1-mediated degradation of NRF2, resulting in a stabilization and a nuclear accumulation of NRF2
Cervix SCC, PRCC, lung AC, lung SCC	c.85G>T	p.D29Y	Missense/pathogenic	Neh2 DLG	Lead to a constitutive activation of NRF2 at E3 ligase recognition sites
HCC, lung SCC, ESCC, SCC-UADT	c.86A>G	p.D29G	Missense/pathogenic	Neh2 DLG	Lead to weaker KEAP1-binding DLG region and inhibit the KEAP1-mediated degradation of NRF2
ccRCC	c.86A>T	p.D29V	Missense/pathogenic	Neh2 DLG	Lead to a constitutive activation of NRF2 at E3 ligase recognition sites
Liver, lung SCC, ESCC, skin, SCC-UADT	c.88C>T	p.L30F	Missense/pathogenic	Neh2 DLG	Affect the binding ability of NRF2 to the KEAP1 dimer with a lower NRF2 ubiquitination and an enhancement of its transcriptional activity into the nucleus
EEA	c.89T>G	p.L30R	Missense/pathogenic	Neh2 DLG	Disrupt the KEAP1/NRF2 interaction due to lack of binding of NRF2 to Kelch domain of KEAP1
Central nervous system	c.91G>A	p.G31R	Missense/pathogenic	Neh2 DLG	Gain-of-function mutation with an increased NRF2 stabilization
ccRCC, lung SCC, ESCC, skin SCC	c.92G>C	p.G31A	Missense/pathogenic	Neh2 DLG	*NFE2L2* mutant with a gain-of-function activity, which is partly resistant to KEAP1-mediated inhibition
ESCC	c.92G>A	p.G31E	Missense/pathogenic	Neh2 DLG	Affect the binding ability of NRF2 to interact with the Kelch domain of KEAP1
SCC-UADT, skin SCC	c.93_95delAGT	p.V32del	In frame/NS	Neh2 DLG	Lead to weaker KEAP1-binding DLG region and inhibit the KEAP1-mediated degradation of NRF2
HCC	c.95T>A	p.V32E	Missense/pathogenic	Neh2 DLG	Mutational target that leads to its aberrant activation
Lung SCC	c.95T>G	p.V32G	Missense/pathogenic	Neh2 DLG	Lead to weaker KEAP1-binding DLG region and inhibit the KEAP1-mediated degradation of NRF2
EEA, lung SCC	c.100C>G	p.R34G	Missense/pathogenic	Neh2	Inhibit the KEAP1-mediated degradation of NRF2, resulting in an increase of its stabilization and nuclear accumulation
Lung SCC, ESCC	c.101G>C	p.R34P	Missense/pathogenic	Neh2	Lead to a constitutive activation of NRF2 at E3 ligase recognition sites
Cervix SCC, lung SCC, ESCC, SCC-UADT	c.101G>A	p.R34Q	Missense/pathogenic	Neh2	Lead to weaker KEAP1-binding DLG region and inhibit the KEAP1-mediated degradation of NRF2
PRCC	c.105_107delAGT	p.V36del	In frame/NS	Neh2	*NFE2L2* mutant is insensitive to KEAP1-mediated degradation (*NFE2L2* mutant is stable even with ectopic expression of KEAP1 occurs)
ESCC, UADT	c.225A>C	p.Q75H	Missense/pathogenic	Neh2	Impair two-site substrate of KEAP1 recognition and inhibit the KEAP1-mediated degradation of NRF2
SCC-UADT	c.229G>A	p.D77N	Missense/pathogenic	Neh2 ETGE	Block the KEAP1-NRF2 binding and inhibit the KEAP1-mediated degradation of NRF2
Liver	c.229G>T	p.D77Y	Missense/pathogenic	Neh2 ETGE	Affect the NRF2 binding to Kelch domain surface of the KEAP1
Lung SCC	c.230A>C	p.D77A	Missense/pathogenic	Neh2 ETGE	Block the KEAP1-NRF2 binding and inhibit the KEAP1-mediated degradation of NRF2
HCC, lung SCC, ESCC	c.230A>G	p.D77G	Missense/pathogenic	Neh2 ETGE	*NFE2L2* mutant with a gain-of-function activity, partly resistant to the KEAP1-mediated inhibition
Lung SCC,ESCC	c.230A>T	p.D77V	Missense/pathogenic	Neh2 ETGE	Compromise the association of NRF2 with KEAP1-DC and inhibit the KEAP1-mediated degradation of NRF2
ESCC	c.232G>A	p.E78K	Missense/pathogenic	Neh2 ETGE	*NFE2L2* mutant with gain-of-function activity, which is partly resistant to the KEAP1-mediated inhibition
Lung SCC, ESCC	c.235G>A	p.E79K	Missense/pathogenic	Neh2 ETGE	Reduce the ability of NRF2 to interact with KEAP1 and inhibit the KEAP1-mediated degradation of NRF2, thus promoting the nuclear localization and transcriptional activity of NRF2
Cervix SCC, lung AC, lung SCC, SCC-UADT	c.235G>C	p.E79Q	Missense/pathogenic	Neh2 ETGE	Compromise the association of NRF2 with KEAP1-DC and inhibit the KEAP1-mediated degradation of NRF2
HCC, SCC-UADT	c.236A>G	p.E79G	Missense/pathogenic	Neh2 ETGE	Block the KEAP1-NRF2 binding and inhibit the KEAP1-mediated degradation of NRF2
HCC, ESCC	c.238A>C	p.T80P	Missense/pathogenic	Neh2 ETGE	*NFE2L2* mutant with a gain-of-function activity, partly resistant to the KEAP1-mediated inhibition
HCC, UADT	c.239C>T	p.T80I	Missense/pathogenic	Neh2 ETGE	Impair the two-site substrate recognition of KEAP1 and inhibit KEAP1-mediated degradation of NRF2
Lung SCC	c.239C>G	p.T80R	Missense/pathogenic	Neh2 ETGE	Reduce the ability of NRF2 to interact with KEAP1
PRCC, lung SCC, ESCC	c.239C>A	p.T80K	Missense/pathogenic	Neh2 ETGE	Compromise the association of NRF2 with KEAP1-DC. Also inhibit KEAP1-mediated degradation of NRF2 and promote nuclear localization and transcriptional activity of NRF2
HCC, lung SCC	c.241G>A	p.G81S	Missense/pathogenic	Neh2 ETGE	*NFE2L2* mutant with gain-of-function activity, with an increased NRF2 stabilization due to alteration of the binding ability to KEAP1
Breast, lung AC, lung SCC, ESCC	c.242G>A	p.G81D	Missense/pathogenic	Neh2 ETGE	*NFE2L2* mutant with a gain-of-function activity, partly resistant to the KEAP1-mediated inhibition
HCC, lung SCC, ESCC	c.242G>T	p.G81V	Missense/pathogenic	Neh2 ETGE	Block the KEAP1-NRF2 binding and inhibit the KEAP1-mediated degradation of NRF2
HCC, lung AC, lung SCC, ESCC	c.244G>C	p.E82Q	Missense/pathogenic	Neh2 ETGE	Block the KEAP1-NRF2 binding and inhibit the KEAP1-mediated degradation of NRF2
PRCC, HCC, lung SCC, UADT	c.245A>G	p.E82G	Missense/pathogenic	Neh2 ETGE	Stabilize NRF2 by disrupting its ability to bind KEAP1 although the NRF2 transcriptional activity remains unchanged
ESCC	c.245A>T	p.E82V	Missense/pathogenic	Neh2 ETGE	Affect the repressive activity of KEAP1 on NRF2 signaling
HCC, lung AC, ESCC	c.246A>C	p.E82D	Missense/pathogenic	Neh2 ETGE	Impair the two-site substrate recognition of KEAP1 and inhibit KEAP1-mediated degradation of NRF2

Cosmic (Catalogue of Somatic Mutations in Cancer) database IP (http://cancer.sanger.ac.uk/cosmic/gene/analysis?ln=NFE2L2#variants). AC: adenocarcinoma; ccRCC: clear cell renal cell carcinoma; DC: C-terminal *β*-propeller domain; DLGex: extended DLG motif; EEA: endometrioid endometrial adenocarcinoma; ESCC: esophageal squamous cell carcinoma; HCC: hepatocellular carcinoma; PRCC: papillary renal cell carcinoma; SCC: squamous cell carcinoma; SCLC: small cell lung cancer; UADT: upper aerodigestive tract; NS: not specified.

**Table 3:** Effect of *KEAP1/NRF2* expression by promoter methylation in human cancers (tissues and cell lines) and correlation with clinical patients' outcomes.

Molecular target	Cancer model	Methylation status	Functional effects	Clinical outcome	Refs
*NRF2*	Prostate cancer cell line (LNCaP) and tissues (*n* = 27)	Hypermethylation	↓ *NRF2* mRNA ↓ *HO-1* mRNA and protein ↓ *NQO1* mRNA and protein	Increment of Gleason score from normal to advanced stage prostate cancer	[121]
	Human colon cancer cells (SNU-C5)	Hypomethylation	↑ *NRF2* mRNA ↑ *HO-1* mRNA and protein	NA	[59, 60]

*KEAP1*	Lung cancer cell lines (SPC-A1, A549, and NCI-H460) and tissues (*n* = 5)	Hypermethylation	↓ *KEAP1* mRNA ↑ NRF2 protein ↑ *HO-1* mRNA and protein ↑ *GSH* mRNA and protein	NA	[48,116, 122]
	NSCLC tissues (*n* = 47)	Hypermethylation	↓ *KEAP1* mRNA	Worst prognosis associated to *KEAP1* double alterations	[52]
	Malignant gliomas (*n* = 86)	Hypermethylation	↓ *KEAP1* mRNA	Lowest risk to progress in patients treated with radiotherapy and temozolomide	[51]
	Primary breast cancers (*n* = 102) and preinvasive breast lesions (*n* = 14)	Hypermethylation	↓ *KEAP1* mRNA	Worse prognosis in triple-negative phenotype and reduced risk of relapse in patients treated with EC/D chemotherapy	[53]
	Human colorectal cancer cell lines (*n* = 10) and tissues (*n* = 40)	Hypermethylation	↓ *KEAP1* mRNA ↑ NRF2 protein ↑ *NQO1* mRNA ↑ *AKR1C1* mRNA	NA	[55]
	Prostate cancer cell DU-145	Hypermethylation	↓ *KEAP1* mRNA ↑ *NRF2* mRNA ↑ *NQO1* mRNA ↑ *HO-1* mRNA ↑ *GCLC* mRNA	NA	[49]
	ccRCC tissues (*n* = 37) TCGA dataset (*n* = 481)	Hypermethylation	↓ *KEAP1* mRNA	Worse overall survival (OS) and association with increased of tumor grading	[54]

*AKR1C1*: aldo-keto reductase family 1 member C1; ccRCC: clear renal cell carcinoma; *GCLC*: glutamate-cysteine ligase catalytic subunit; *GSH*: glutathione; *HO-1*: heme oxygenase-1; *KEAP1*: Kelch-like ECH-associated protein 1; *NQO1*: NAD(P)H-quinone oxidoreductase 1; *NRF2*: nuclear factor erythroid 2-related factor 2; NSCLC: non-small-cell lung cancer; NA: not applicable.

**Table 4:** Main miRNAs interacting with *KEAP1* and *NRF2* and downstream effects observed in cancer cells.

Target	miRNA ID	Functional validations			
		Downstream effects	Cancer model	Methods	Refs
*NRF2*	↑ hsa-miR-28	↓ *NRF2*	Human breast cancer cells	Luciferase assay, qRT-PCR, WB, and coimmunoprecipitation	[76]
	↓ hsa-miR-155	↑ *NRF2*, ↑ *HO-1*, ↑ *GSH*, ↑ *NO*, ↑ *SOD*, ↑ colony formation, ↑ arsenite resistance	Human bronchiolar epithelial cells	Mimic and miRNA inhibitor transfection, qRT-PCR, WB, measurements of ROS and enzymatic activity, cell cycle, cell migration, and colony formation assays	[78]
	↑ hsa-miR-144	↓ *NRF2*, ↓ *GSH*, ↓ cell viability, ↑ apoptosis, ↓ 5-FU resistance	Leukemia cells, human hepatocellular cancer cells, neuroblastoma cells	Luciferase reporter assay, cell viability assay, ROS and enzymatic activity measurements, qRT-PCR, WB	[82]
				MicroRNA microarray analysis, luciferase reporter assay, qRT-PCR, WB, ELISA	[83]
				Luciferase reporter assay, drug sensitivity assay, qRT-PCR, WB	[84]
	↓ hsa-miR-93	↑ *NRF2*, ↑ mammosphere formation, ↓ apoptosis, ↑ DNA damage	Rat breast cancer cells	siRNA (small interfering RNA) transfection, qRT-PCR, WB, clonogenic cell survival assay, mammosphere formation assay, cell migration, and cell apoptosis assays	[85]
	↑ hsa-miR-153	↓ *NRF2*, ↓ *GCLC*, ↓ *GSR*, ↓ cell migration, ↓ cell invasion	Neuroblastoma cells, breast cancer cells, oral squamous cell carcinoma	miRNA mimic and inhibitor transfection, cell proliferation assay and colony forming assays, cell migration analysis, cell cycle analysis, measurement of ROS and enzymatic activity, qRT-PCR, WB	[87]
				MicroRNA microarray analysis, luciferase reporter assay, qRT-PCR, WB, cell migration, and invasion assays	[88]
	↑ hsa-miR-27 ↑ hsa-miR 142	↓ *NRF2*, ↓ *GCLC*, ↓ *GSR*	Neuroblastoma cells	Transient transfection, WB, qRT-PCR, measurement of ROS, and enzymatic activity	[86]
	↑ hsa-miR-340	↓ *NRF2*, ↓ cisplatin resistance	Esophageal cancer cells	MicroRNA microarray analysis, luciferase reporter assay, drug sensitivity assay, qRT-PCR, WB	[89]
	↑ hsa-miR-507 ↑ hsa-miR-634 ↑ hsa-miR-450 ↑ hsa-miR-129	↓ *NRF2*, ↓ *ME1*, ↓ cell growth, ↓ cisplatin resistance	Esophageal cancer cells	Measurement of ROS, qRT-PCR, WB, siRNA, drug sensitivity assays	[90, 91]

*KEAP1*	↑ hsa-miR-141	↓ *KEAP1*, ↑ *NRF2*, ↑ *HO-1*, ↓ apoptosis, ↑ cisplatin resistance, ↑ 5-FU resistance	Ovarian cancer cells, hepatocellular carcinoma	MicroRNA microarrays analysis, luciferase reporter assay, qRT-PCR, WB, apoptosis assay	[92]
				MicroRNA microarray analysis, miRNA mimic and inhibitor transfection, luciferase reporter assay, qRT-PCR, WB, cell viability assay, apoptosis assay, drug sensitivity assay	[93]
	↑ hsa-miR-200	↓ *KEAP1*, ↑ *NRF2*	Breast cancer cells, esophageal cancer cells	Luciferase reporter assay, qRT-PCR, mRNA stability assay, WB, ChIP, anchorage-independent cell growth assay, IHC	[60]
				Transfection reporter assay, qRT-PCR, ChIP	[95]
	↑ hsa-miR-7	↓ *KEAP1*, ↑ *NRF2*, ↑ *GSH*	Neuroblastoma cells	Cell viability assay, LC-MS/MS, qRT-PCR, WB, enzymatic activity measurements	[96]
	↑ hsa-miR-432	↓ *KEAP1*, ↑ *NRF2*, ↑ chemoresistance	Esophageal cancer cells	CRISPR/Cas9 system, cell survival assay, siRNAs, luciferase reporter assay, qRT-PCR, WB, mRNA stability assay, ChIP	[98]

*GCLC*: glutamate-cysteine ligase catalytic subunit; *GSH*: glutathione; *GSR*: glutathione reductase; *HO-1*: heme oxygenase-1; *KEAP1*: Kelch-like ECH-associated protein 1; *ME1*: malic enzyme 1; *NO*: nitric oxide; *NRF2*: nuclear factor erythroid 2-related factor 2; *SOD*: superoxide dismutase; *5-FU*: 5-fluorouracil; WB: Western blot; FC: flow cytometry; IHC: immunohistochemistry; LC-MS/MS: liquid chromatography tandem mass spectrometry; MTT: (3-(4,5-dimethylthiazol-2-yl)-2,5-diphenyl tetrazolium bromide) assay; qRT-PCR: quantitative real-time PCR.
